# Analysis of Venous Insufficiency Risk Factors and Demographic Characteristics among Nurses: An Analytical Cross-Sectional Study

**DOI:** 10.3390/medicina60091498

**Published:** 2024-09-13

**Authors:** Sevcan Avcı Işık, Elif Budak Ertürk, Hakkı Tankut Akay, Azize Karahan, Denizhan Akpınar, Arif Okay Karslıoğlu

**Affiliations:** 1 Department of Nursing, Faculty of Health Science, Başkent University, 06790 Ankara, Türkiye; elifbudakerturk@gmail.com (E.B.E.); kazize@baskent.edu.tr (A.K.); 2 Department of Cardiovascular Surgery, Faculty of Medicine, Başkent University, 06790 Ankara, Türkiye; tankutakay@gmail.com (H.T.A.); denizhanakpinar@baskent.edu.tr (D.A.); aokaykarslioglu@baskent.edu.tr (A.O.K.)

**Keywords:** chronic venous insufficiency, varicose veins, risk factors, nurse, CEAP

## Abstract

*Background and Objectives:* Chronic venous insufficiency negatively affects the quality of life and reduces the job performance of nurses, who are important components of the healthcare system. The aim of this study was to assess the risk factors of venous insufficiency according to demographic characteristics among nurses working at a foundation university hospital. *Materials and Methods:* This study used an analytical cross-sectional approach. The sample consisted of 100 nurses working at a foundation university hospital in a metropolitan city of Turkey. Data were collected using a demographic characteristics form, VEINESQOL/Sym, and a CEAP classification form. The condition of varicose veins among the nurses was diagnosed by a cardiovascular surgeon using Doppler ultrasonography. *Results:* The prevalence of chronic venous insufficiency (CVI) among nurses was 65%, with 48% at a C1 level according to the CEAP classification. CVI was higher among those with chronic diseases (*p* = 0.027) and those who had pregnancy (*p* = 0.021). In addition, the risk of CVI (+) was 7.68 times higher among those aged older than 26.5 years and 36.14 times higher for women (*p* < 0.001). A 0.9-fold increase in the risk of CVI (+) among nurses produced a one-unit decrease in venous-insufficiency-related quality of life (*p* = 0.006, OR = 0.94, 95% CI:(0.896–0.982)). *Conclusions:* The prevalence of CVI among nurses was found to be high, especially among women, those with chronic diseases, and pregnant individuals. In this context, it is recommended to implement risk screening and prevention education programs for CVI among nurses.

## 1. Introduction

Chronic venous insufficiency (CVI) is a condition resulting from obstruction, valvular insufficiency, muscle pump dysfunction, or a combination of these factors. CVI is a very common problem, affecting more than 25 million individuals in the United States and more than six million individuals with more advanced venous disease [1]. Obstruction is present in 10% and valve insufficiency in 90% of patients. Moreover, CVI can occur in both superficial and deep leg veins [2]. The prevalence of CVI is reported to be 1–40% in women and 1–27% in men. In addition, the annual increase rate was reported to be 2.6% among women and 1.9% among men [3].

In chronic venous insufficiency, reflux, which develops as a result of damage or dysfunction of the valves, causes venous pressure to increase. The disruption of venous circulation triggers leukocyte–endothelial interactions and produces a chronic inflammatory state. In advanced stages of venous insufficiency, atrophic changes in the skin and venous ulcers develop due to occlusive inflammatory changes in venules and arterioles, vascular fibrosis, and degeneration [2,4,5]. Patients with CVI have pain and edema that increase with standing due to the aforementioned pathologic changes. This disease may also include varicose veins, pigmentation, lipodermatosclerosis, and venous scarring. Stasis ulcers occur due to the dilatation of superficial veins, rupture of fine veins, and obstructions caused by thrombosis in deep veins [5]. The appearance of skin among those with CVI is dry, cracked, and itchy [2,3,4].

The most significant complication of chronic venous insufficiency is venous ulcers. The frequency of venous ulcers, which represent the most advanced stage according to CEAP classification, is 0.3%. In previous studies, perforating venous insufficiency was also reported in around 60% of patients with venous ulcers [3]. Varicose veins, which are a manifestation of chronic venous insufficiency, represent an important health problem due to their high prevalence and significantly negative impact on the labor force and quality of life, as well as their epidemiological and socioeconomic consequences [6].

The most important risk factors for chronic venous insufficiency are age, gender, genetic predisposition, prolonged standing, obesity, sedentary lifestyle, irregular bowel movements, high systolic blood pressure, smoking, pregnancy, history of thrombophlebitis, lower extremity trauma, and flat feet [2,4,5,7,8].

Nursing is considered to be a risky occupation in terms of chronic venous insufficiency because it requires prolonged standing [6]. In a study conducted among 203 nurses from three different hospitals in Iran, the prevalence of varicose veins among nurses was determined to be 72.4%, with women having a higher prevalence than men (77.9% vs. 56.9%). Other risk factors in the study included increased working years, long working hours, overtime, and body mass index [9]. The prevalence of varicose veins among 414 nurses working at a university hospital was determined to be 16.18%. In this study, an older age and working more than four hours while standing were determined to be risk factors for venous insufficiency [6]. In another study conducted on nurses, the prevalence of venous insufficiency by gender was determined to be 88.2% among female nurses and 11.8% among male nurses [10]. In a study on 181 nurses conducted by Shakya et al. (2020) [11], 46% had varicose veins, with a significant relationship between an increase in the standing time of the nurses and the incidence of varicose veins.

Chronic venous insufficiency affects the quality of life and decreases the work performance of nurses. A previous study on 207 healthcare workers diagnosed with CVI found that the pain experienced by healthcare workers negatively affected their quality of life [12]. Another study examining CVI’s impacts on quality of life among nurses determined that their quality-of-life levels were moderate [13]. Since nurses are the main components of healthcare systems, such situations cause disruptions to health services and require economic resources to be spent on treatment [14]. This study aims to analyze the risk factors and demographic characteristics of venous insufficiency among nurses working in a foundation university hospital. To the best of our knowledge, no previous study in the literature has determined and verified the prevalence of CVI among nurses using expert evaluations with both physical and Doppler Ultrasonography (USG) alongside an analysis of risk factors and quality of life. Therefore, this study is novel because it is the to investigate the risk factors of venous insufficiency among nurses, assess those factors with diagnostic methods, and simultaneously investigate the quality of life of nurses related to venous insufficiency. 

## 2. Materials and Methods

### 2.1. Study Design and Participation

The present study used analytical cross-sectional analysis to assess the risk factors and demographic characteristics of venous insufficiency among nurses working in a foundation university hospital. The Strengthening the Reporting of Observational Studies in Epidemiology (STROBE) statement was used as a framework to report the study design and findings.

The present study was conducted at a foundation university hospital in a metropolitan city in Turkey between 24 May and 24 June 2023. In total, 271 nurses were working in the hospital during the time period of the study. Here, nurses working in administrative positions in the directorate of nursing services, charge nurses, training nurses, and outpatient clinic nurses work between 08:00 and 16:00; ward nurses and nurses in other departments work between 08:00 and 20:00 and between 20:00 and 08:00; and operating room nurses work between 08:00 and 16:00 and between 16:00 and 08:00. Nurses work at least 48 h a week, but their weekly working hours can extend up to 60 h.

The population of the study consisted of 271 nurses working at Başkent University Ankara Hospital. N was used to calculate the sample size required for estimating the ratio of the population (N = 271), as follows:

$$n=\frac{N\;*\;t^{2}*p*\left(1-p\right)}{\;{\left(N-1\right)*\;d}^{2}+t^{2}*p*\left(1-p\right)}$$

Accordingly, the minimum sample size required for this study was determined to be α = 0.05, d = 0.075, and *p* = 0.50, totaling 100 people at a 95% confidence level. This proportion represents the maximum sample size to be obtained using the relevant Type I error probability and the amount of tolerance. Those with current pregnancy throughout the study, deep vein thrombosis (DVT), cancer, leg paresis, peripheral arterial disease, phlebitis, and very recent surgery or anesthesia, as well as those on sick leave, were excluded from the study. All nurses who agreed to participate in the study and who did not meet the exclusion criteria constituted the sample. The study was ultimately completed with 100 nurses.

### 2.2. Data Collection Forms

In data collection, we used the demographic characteristics of the nurses [4,10,13,15,16,17] alongside the venous insufficiency risk factors determination form, CEAP form, and Venous Insufficiency Epidemiologic and Economic Study—Quality of Life Questionnaire (VEINESQOL/Sym) to determine the level of venous insufficiency.

### 2.3. Demographic Characteristics of Nurses and Form for Determining Venous Insufficiency Risk Factors

This form consists of two parts. The first part (self-completed) includes questions on age, gender, height, weight, body mass index (BMI), marital status, educational level, occupation, chronic disease status, number of pregnancies, smoking/alcohol use, exercise, bowel movements, family history of varicose veins, and footbed. The second part (self-completed) includes questions on years of service; unit of employment; position in the unit; shift characteristics; number of overtime hours worked per week; and sitting, standing, and walking patterns.

### 2.4. Standard CEAP Form 

When chronic venous insufficiency is suspected in the physical examination of patients presenting to the clinic due to complaints, a definitive diagnosis is made using Doppler USG, a non-invasive test [2,3,4]. For venous insufficiency, CEAP classification is used to define the disease and determine its clinical severity. Here, C stands for clinical findings, E for etiology, A for anatomy, and P for pathophysiology. Depending on the presence of specific clinical signs, which are associated with increasing clinical severity but may or may not be symptomatic, chronic venous insufficiency can be classified from C0 (no symptoms) to C6 (venous ulceration) [18]. 

Clinical Classification

C0: no visible or palpable signs of venous disease
C1: telangiectasias or reticular veins
C2: varicose veins
C3: edema
C4a: pigmentation and eczema
C4b: lipodermatosclerosis or atrophie blanche
C5: healed
C6: active venous ulcer

### 2.5. The Venous Insufficiency Epidemiological and Economic Study (VEINESQOL/Sym) Questionnaire

The Venous Insufficiency Epidemiological and Economic Study (VEINESQOL/Sym) Questionnaire is a disease-specific scale developed to assess the quality of life and symptoms among patients with venous insufficiency. This scale was developed by Donna Lamping et al. [19] specifically for the routine follow-up of venous disease outcomes. The Turkish validity and reliability of the scale was developed by Çırak et al. [20]. VEINESQOL/Sym consists of a total of 26 items. The total VEINESQOL/Sym score provides a minimum of 24 and a maximum of 112 points. Low scores indicate a poor quality of life, and high scores indicate improved quality of life. Previously, the Cronbach’s alpha coefficient was calculated as 0.96 for VEINESQOL and 0.94 for VEINES-Sym [20]. In the present study, this value was calculated as 0.81 for VEINES-QOL and 0.85 for VEINES-Sym.

### 2.6. Data Collection Process

The forms given to the nurses required approximately 10 min to fill out. Then, the presence and clinical severity of varicose veins were determined by a specialist cardiovascular surgeon using the standard CEAP form. The specialist doctor also measured and certified the CVI via Doppler USG. The C0 group was evaluated as CVI (−), and the C1, C2, and C3 groups were evaluated as CVI (+). These measurement and evaluation processes were used for all nurse participants.

### 2.7. Ethical Process

After obtaining permission from the Medical and Health Sciences Research Board (KA23/109, Date: 22 March 2023) and the Ethics Committee of the University Hospital where the study was conducted, we obtained permission from the chief physician of the hospital. In addition, written and verbal consent was obtained from the nurses who agreed to participate in the study. 

### 2.8. Statistical Analysis

Statistical analyses were performed using SPSS v25 (SPSS for Windows, Chicago, IL, USA, September 2012; License Number: 1093910). Mean and standard deviation values were given when quantitative variables were presented, and frequency and percentage values were given when qualitative variables were presented. The Shapiro–Wilk test was used to evaluate whether the variables fit the normal distribution. When quantitative variables were evaluated between two groups, ‘Student’s *t*-test’ was used under the assumption of a normal distribution. Pearson’s Chi-square test or Fisher’s exact test were used to compare categorical variables. Variables that may be risk factors for varicose veins were included in univariate logistic regression analysis. Variables found to be significant in univariate logistic regression analysis and independent variables with a significance value below 0.25 were included in multiple logistic regression analysis. The forward elimination method was used to find the best estimated model. Variables with *p* value below 0.05 were considered significant.

## 3. Results

The mean age of the nurses who participated in the study was 31.14 ± 8.12 years; 84% were female, 60% were single, and 56% had finished their undergraduate education. In addition, 59% of the nurses had a normal BMI. Forty-two percent of the participants smoked cigarettes, with an average of 7.95 ± 5.16 years of smoking and 11.52 ± 6.25 cigarettes per day. In total, 28% of the nurses stated that they had never given birth, 46.4% of those who had given birth had given birth once, 79% did not exercise regularly, and 81% had regular bowel movements. Moreover, 18% of the nurses had a chronic disease and 9% had been previously diagnosed with venous insufficiency; those diagnosed with venous insufficiency had received medication (5%), venous compression therapy (3%), sclerotherapy (5%), and surgical treatment (3%). Notably, 38% had someone in their family diagnosed with venous insufficiency. In addition, 94% had a normal footbed.

A statistically significant relationship was found between gender (*p* < 0.001), having a chronic disease (*p* = 0.027), and pregnancy status (*p* = 0.021) when comparing the CVI (+) group with the CVI (−) group according to all demographic characteristics. CVI was found to be higher among women, those with chronic diseases, and those who were pregnant (Table 1).

Although not specified in the table, 38% of the nurses worked in the ward, 36% in intensive care, and 68% as ward nurses, with a mean duration of practice totaling 9.5 ± 8.2 years. In addition, 65% were assigned the 08.00–20.00 shift and worked overtime for an average of 7.08 ± 9.02 h per week. Of the nurses who participated in the study, 52% stated that they walked more than 6 h per day, 47% stated that they stood more than 6 h per day, and 51% stated that they sat for less than 2 h per day.

**Figure 1:**
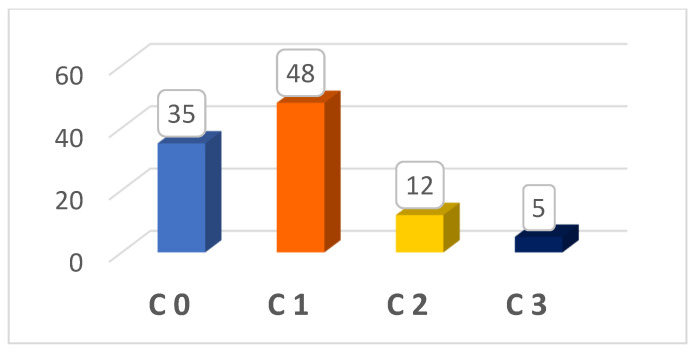
CEAP classification of nurses.

A significant difference was determined according to the unit of work (*p* = 0.021) when we compared the CVI (+) group with the CVI (−) group according to occupational characteristics. No statistically significant difference was found for the other variables (Table 2). Based on Doppler USG, 48% of the nurses were classified as C1, 35% as C0, 12% as C2, and 5% as C3 CEAP (Figure 1). Accordingly, 65% of the nurses had CVI (+) and 35% had CVI (−).

**Figure 2:**
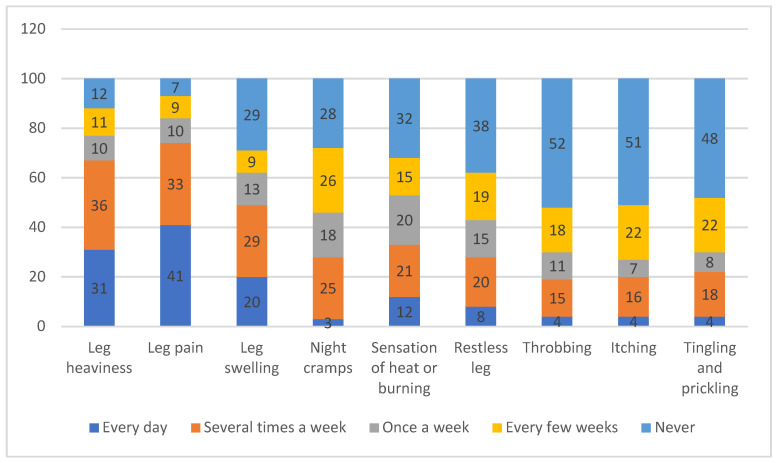
The distribution of complaints.

Based on the physical examination, the mean circumference below the right knee was 36.01 ± 3.77 cm, while that below the left knee was 36.06 ± 3.76 cm; the mean circumference above the right ankle was 24.76 ± 2.72, while that above the left ankle was 24.72 ± 2.82 (data not included in the table). Nurses reported the most common complaints as leg pain, leg heaviness, leg swelling, and night cramps (Figure 2).

The mean item total score of VEINESQOL/Sym was 78.26 ± 13.70. All variables were compared with VEINESQOL/Sym. The mean VEINESQOL/Sym item total score was found to be lower in females (*p* < 0.001), those with chronic disease (*p* = 0.020), those with irregular bowel movements (*p* = 0.007), those with a family history of CVI (*p* = 0.018), and those with CVI (+) (*p* < 0.001) (Table 3).

The risk of varicose veins was 7.68 times higher among nurses older than 26.5 years than among those younger than 26.5 years (*p* = 0.001, OR = 7.68, 95% CI:(2.33–25.32)). Moreover, the risk of CVI (+) was 36.14 times higher for women than for men (*p* = 0.002, OR = 36.14, 95% CI:(3.835–340.54)). A 0.9-fold increase in the risk of CVI (+) among nurses yielded a 1 unit decrease in venous insufficiency-related quality of life (*p* = 0.006, OR = 0.94, 95% CI:(0.896–0.982)). The correct classification rate for this model was 87% (Table 4).

## 4. Discussion

In this study, we determined the risk factors and demographic characteristics of venous insufficiency among nurses working at a foundation university hospital.

Older age was previously defined as an important risk factor for CVI^8^. In our study, an age of 26.5 years was determined to be the threshold value, and the risk of CVI was found to be 7.68 times higher among those aged 26.5 years than among those younger than 26.5 years (Table 4). Similarly, Abou-ElWafa et al. (2020) [15] reported that the incidence of CVI was significantly higher among nurses older than 25 years. In another study conducted with nurses, an increase in age (OR = 1.06, 95% confidence interval (CI) = 1.03–1.10) was identified as a significant risk factor for venous insufficiency [6], possibly because aging is associated with the weakening of and damage to venous valves, thereby facilitating the development of varicose veins [4,21].

In this study, we determined the female gender to be an important risk factor in the development of CVI. Indeed, this risk was 36.14 times higher in women than in men (Table 4). Many studies in the literature have also identified the female gender as a risk factor for CVI [8,10,13,16]. The predisposition of women to CVI (+) may be due to the effects of estrogen and progesterone, as varicose veins in women were previously associated with increased estrogen and progesterone receptors in all tunica layers of the vessel wall. Estrogen is known to decrease venous tone and increase distention in women due to its relaxant effects on smooth muscles and collagen fibers [22]. 

CDV is known to be associated with diseases of the cardiovascular system (hypertension, heart failure, etc.), diabetes mellitus, and some diseases of the skeletal system, such as muscular diseases [8,10,23]. Varicose veins are a symptom of persistent venous hypertension or high blood pressure in the veins. High blood pressure in the veins irreversibly destroys the valves and weakens the vein walls. This disease can cause swelling, pain, heaviness, fatigue, and other symptoms such as leg ulcers and blood clots [7,10]. The mechanism of venous stasis and inflammation in CVI can also damage subchondral areas, leading to cartilage loss and osteoarthritis [24]. In our study, 88.9% of those with chronic diseases had CVI (+), and this result was statistically significant (*p* = 0.027) (Table 3). Although most of the nurses were diagnosed with rheumatologic diseases, 3% had hypertension and 4% had diabetes. 

Pregnancy is another risk factor for CVI due to increased progesterone levels and vasodilation of blood vessels with valvular insufficiency, as well as uterine wall dilation, which increases pressure on vessels in the pelvis and lower extremities [4,6]. Most of the nurses in our study who gave birth were CVI (+) (*p* = 0.021) (Table 3). This result agrees with the literature, and pregnancy was determined to represent a significant risk factor for CVI [9,14].

The prevalence of CVI among nurses in our study was found to be 65% based on Doppler USG and a physical examination. Although only 9% of the nurses stated that they had been previously diagnosed with venous insufficiency, the prevalence in this study was high. Some studies in the literature employed nurses’ reports of CVI prevalence both with and without using a diagnostic method. In a study conducted on 51406 nurses working in 311 hospitals in China, 40.9% of the nurses reported that they had varicose veins [25]. Similarly, a study conducted in India reported the prevalence of CVI among nurses to be 24% [26]. In a different study conducted on nurses, the prevalence of CVI was determined to be 15.8% [10]. In a cross-sectional study conducted on 366 nurses in Saudi Arabia, the rate of those diagnosed with varicose veins was 11% [16]. In a previous study, the prevalence of CVI among nurses was found to be 16.2% based on bidirectional ultrasonography, one of the diagnostic methods applied in the present work [6]. In a study evaluating 201 nurses for VCI risks using Doppler ultrasound, the prevalence of varicose veins was found to be 18.4% [15]. In our study, the rate of nurses with CVI (+) was found to be higher than that in other studies, possibly because the majority of nurses in our country are women and have longer working hours. Although 9% of the nurses stated that they were diagnosed with CVI (+), the number of patients diagnosed via the cardiovascular surgeon’s Doppler USG evaluation and physical examination was remarkably higher. This discrepancy may be due to the fact that nurses’ CVI awareness, screening programs, and the application of preventive measures, such as using compression stockings, are not sufficient. 

Approximately 25% of adults in the United States are affected by chronic venous disease, which leads to a significant decrease in quality of life (QOL) and associated disease burden, as well as a loss of work productivity [27,28]. In a study conducted by Yıldız Şahin and Karaman Özlü (2021) [29] on 312 patients, the mean quality of life associated with the venous insufficiency score of the patients was 74.95 ± 12.86, which was considered moderate. A study conducted among healthcare professionals determined that the mean quality of life associated with the venous insufficiency score of the nurses was 63.1 ± 11.5, which again reflects a moderate level [13]. Our study also determined that the quality of life among nurses related to venous insufficiency was moderate. However, the quality of life of nurses with CVI (+) was lower and decreased as the risk of CVI (+) increased (Table 3 and Table 4). Overall, the findings in the literature support the findings of our study. 

Although CVI-related quality of life is affected by many conditions, studies on nurses are very limited. In our study, we determined that women (*p* < 0.001), those with chronic diseases (*p* = 0.020), those with irregular bowel movements (*p* = 0.007), and those with a family history of CVI (*p* = 0.018) had a lower mean VEINESQOL/Sym item total score (Table 3). The reasons for this result may be that nurses experienced symptoms such as leg pain, leg heaviness, leg swelling and night cramps, as reported in the present study (Figure 2). Another study conducted among healthcare workers further determined that quality of life was significantly lower among women, those of an older age, those with higher total working years, and those who were very mobile at work [13]. In a study examining 3008 participants in France, those with CVI symptoms were found to have lower quality-of-life levels than those of participants at risk for CVI (*p* < 0.001) [30]. A meta-analysis comparing the quality of life of patients with mild and moderate CVI (+) found that the quality of life for patients with severe CVI (+) was worse in physical areas [31]. In our study, those without regular bowel movements had lower quality-of-life scores (*p* = 0.007). Indeed, constipation is one of the conditions that triggers chronic venous insufficiency, which negatively affects quality of life [17,32]. In another study, the symptoms experienced by the patients were mostly pain, swelling, restlessness in the legs, heaviness in the legs, night cramps, and itching; however, unlike the present study, quality of life was not associated with any condition (*p* > 0.05) [33]. Another interesting finding in our study was the lack of a correlation between body mass index (BMI) and chronic venous insufficiency. Although overweight nurses also suffered from CVI, this number was not statistically significant (14 and 37.8% versus 23 and 62.2%, *p* = 0.177) and may be due to the time spent at work or age. The number of years working as a nurse also emerged an independent risk factor that did not always correlate with the age. For instance, an overweight nurse who has worked for two years may not have developed CVI, whereas an underweight nurse who has worked for 10 years may have developed CVI.

### Strengths and Weaknesses

Nurses’ self-reports were only one component of this study. To confirm the diagnosis of VCI(+), a cardiovascular surgeon performed a physical examination and evaluation with Doppler ultrasound. The results of the nurses’ assessments were then compared and evaluated with their self-reported quality of life. To the best of our knowledge, no other study in the literature has determined the prevalence of VCI among nurses based on an expert evaluation alongside an analysis of risk factors and quality of life. In this respect, the present study is considered to be of significant scientific benefit. However, since this study was a single-center study, it may not represent the prevalence of VCI(+) in Turkey. Accordingly, it is recommended to conduct a prevalence study using a larger sample group.

## 5. Conclusions

According to the results of the present study, the prevalence of CVI is high among nurses. While age and gender constitute a high risk for the occurrence of CVI within this group, chronic disease and pregnancy status were also found to be important variables in the occurrence of CVI. In addition, nurses with CVI (+) had a lower quality of life than those with CVI (−). Ultimately, the prevalence of CVI is high among nurses, who spend the most time with patients in the healthcare system and work long hours on their feet, exacerbating this development and the impact of this disease. Accordingly, it is recommended to identify risk factors, potential lifestyle changes, and prevention programs to reduce or prevent venous insufficiency among nurses who are new to the profession. In addition, screening nurses for venous insufficiency in certain periods will contribute to the prevention of this disease by increasing awareness.

## Figures and Tables

**Table 1:** Comparative analysis of demographic variables of CVI (+) and CVI (−) groups (n = 100).

		CVI (−) n (%)	CVI (+) n (%)	*p*
**Gender**	Female	20 (23.8%)	64 (76.2%)	**0.001** ^a^
	Male	15 (93.8)	1 (6.3)	
**BMI**	Underweight (<18.5)	3 (75%)	1 (25%)	0.177 ^b^
	Normal (18.5–25)	18 (30.5%)	41 (69.5)	
	Overweight (>25)	14 (37.8)	23 (62.2)	
**Marital status**	Married	10 (25%)	30 (75%)	0.134 ^a^
	Single	25 (41.7%)	35 (58.3%)	
**Education status**	Highschool	12 (34.3%)	23 (65.7%)	0.516 ^b^
	Undergraduate	22 (39.3%)	34 (60.7%)	
	Graduate	1 (11.1%)	8 (88.9%)	
**Smoking status**	Yes	18 (42.9%)	24 (57.1%)	0.117 ^a^
	No	14 (29.3%)	41 (70.7%)	
Pregnancy status *	Yes	3 (10.7%)	25 (89.3%)	**0.021** ^a^
	No	17 (30.4%)	39 (69.6%)	
**Number of childbirths**	1	2 (15.4%)	11 (84.6%)	0.403 ^b^
	2	1 (8.3%)	11 (91.7%)	
	≥3	0 (0%)	3 (100%)	
Chronic disease status **	Yes	2 (11.1%)	16 (88.9%)	0.027 ^a^
	No	33 (40.2%)	49 (59.8%)	
**Exercise status**	1 h	2 (66.7%)	1 (33.3%)	0.232 ^b^
	2 h	4 (44.4%)	5 (55.6%)	
	≥3 h	3 (33.3%)	6 (66.7%)	
	I don’t exercise regularly	26 (32.9%)	53 (67.1%)	
**Bowel movements**	Regular	30 (28.4%)	51 (52.7%)	0.435 ^a^
	Irregular	5 (26.3%)	14 (73.7%)	
**Footbed**	Pes planus	3 (50%)	3 (50%)	0.420 ^a^
	Normal	32 (34%)	62 (66%)	
**Previous diagnosis of CVI**	Yes	1 (11.1%)	8 (88.9%)	0.155 ^a^
	No	34 (37.4%)	57 (62.6%)	
**Family history of CVI**	Yes	9 (23.7%)	29 (76.3%)	0.084 ^a^
	No	26 (41.9%)	36 (58.1%)	

^a^: Fisher’s Exact Test; ^b^: Linear-by-Linear Association; BMI: Body Mass Index. * Calculated using 84 samples. ** Among those with chronic diseases, 3% had hypertension, 4% had diabetes mellitus, 9% had rheumatologic diseases, and 5% had other diseases.

**Table 2:** Comparative analysis of the professional characteristics of CVI (+) and CVI (−) groups (n = 100).

		CVI (-) n (%)	CVI (+) n (%)	*p*
**Working units**	Nursing management services	4 (40%)	6 (%60)	**0.021** ^a^
	Wards	15 (39.5)	23 (60.5%)	
	Intensive care	16 (44.4%)	20 (55.6%)	
	Operating room/ Emergency room/ Interventional departments	0 (0%)	6 (100%)	
	Policlinics	0 (0%)	10 (100%)	
**Position**	Head nurse	4 (28.6%)	10 (71.4%)	0.456 ^a^
	Ward nurse	28 (41.2%)	40 (58.8%)	
	Outpatient clinic nurse	1 (8.3%)	11 (91.7%)	
	Manager/Assistant manager/Block manager/Supervisor	2 (33.3%)	4 (66.7%)	
**Daily walking time in the working unit**	<2 h	0 (0%)	5 (100%)	0.078 ^a^
	2–4 h	4 (26.7%)	11 (73.3%)	
	4–6 h	10 (35.7%)	18 (64.3%)	
	≥6 h	21 (40.4%)	31 (59.6%)	
**Daily sitting time in the working unit**	<2 h	17 (33.3%)	34 (66.7%)	0.848 ^a^
	2–4 h	14 (36.8%)	24 (63.2)	
	4–6 h	4 (40%)	6 (60%)	
	≥6 h	0 (0%)	1 (10%)	
**Daily standing time in the working unit**	<2 h	1 (33.3%)	2 (66.7%)	0.690 ^a^
	2–4 h	6 (35.3%)	11 (65.7%)	
	4–6 h	10 (30.3%)	23 (69.7%)	
	≥6 h	18 (38.3%)	29 (31.7%)	

^a^: Linear-by-Linear Association.

**Table 3:** Comparison of nurses’ descriptive characteristics and VEINESQOL/Sym (n = 100).

		VEINES QOL			
		n	Mean ± SD	Test	*p*
**Gender**	Female	84	75.76 ± 12.87	−4.580	**<0.001**
	Male	16	91.37 ± 10.15		
**Chronic disease status**	Yes	18	71.50 ± 9.59	−2.365	**0.020**
	No	82	79.74 ± 14.06		
**Bowel movements**	Regular	81	80.03 ± 13.59	2.767	**0.007**
	Irregular	19	70.68 ± 11.65		
**Family history of CVI**	Yes	38	74.13 ± 15.17	−2.416	**0.018**
	No	62	80.79 ± 12.16		
**CVI**	−	35	86.74 ± 13.32	5.081	**<0.001**
	+	65	73.69 ± 11.63		
**VEINESQOL/Sym Total Score**	78.26 ± 13.70				

SD: Standard deviation; CVI: Chronic venous insufficiency.

**Table 4:** Multiple logistic regression analysis with the forward: LR method (n = 100).

	B	S.E.	Wald	*p*	Exp(B)	95% C.I. for EXP(B)	
						Lower	Upper
Age (>26.5)	2.038	0.609	11.204	**0.001**	7.677	2.327	25.321
Gender (Female)	3.587	1.144	9.825	**0.002**	36.140	3.835	340.543
VEINESQOL/Sym	−0.064	0.023	7.547	**0.006**	0.938	0.896	0.982

## Data Availability

Data are available upon reasonable request.
